# Inequalities in children’s mental health before and during the COVID-19 pandemic: findings from the UK Household Longitudinal Study

**DOI:** 10.1136/jech-2022-220188

**Published:** 2023-09-25

**Authors:** Naomi Miall, Anna Pearce, Jamie C Moore, Michaela Benzeval, Michael J Green

**Affiliations:** 1 MRC/CSO Social and Public Health Sciences Unit, School of Health and Wellbeing, University of Glasgow, Glasgow, UK; 2 Institute for Social and Economic Research (ISER), University of Essex, Colchester, UK; 3 Department of Obstetrics and Gynecology, Duke University School of Medicine, Durham, North Carolina, USA

**Keywords:** Health inequalities, EPIDEMIOLOGY, CHILD HEALTH, COVID-19

## Abstract

**Background:**

There are concerns that child mental health inequalities may have widened during the COVID-19 pandemic. We investigated whether child mental health inequalities changed in 2020/2021 compared with prepandemic.

**Methods:**

We analysed 16 361 observations from 9272 children in the population representative UK Household Longitudinal Study. Child mental health was measured using the Strengths and Difficulties Questionnaire (SDQ) at ages 5 and 8 years in annual surveys 2011–2019, and at ages 5–11 years in July 2020, September 2020 and March 2021. Inequalities in cross-sectional SDQ scores among 5 and 8 year olds, before and during the pandemic, were modelled using linear regression. Additionally, interactions between time (before/during pandemic) and: sex, ethnicity, family structure, parental education, employment, household income and area deprivation on mental health were explored.

**Results:**

A trend towards poorer mental health between 2011 and 2019 continued during the pandemic (b=0.12, 95% CI 0.08 to 0.17). Children with coupled, highly educated, employed parents and higher household income experienced greater mental health declines during the pandemic than less advantaged groups, leading to narrowed inequalities. For example, the mean difference in child SDQ scores for unemployed compared with employed parents was 2.35 prepandemic (1.72 to 2.98) and 0.02 during the pandemic (−1.10 to 1.13). Worse scores related to male sex and area deprivation were maintained. White children experienced worse mental health than other ethnicities, and greater declines during the pandemic.

**Conclusion:**

Mental health among UK 5 and 8 year olds deteriorated during the pandemic, although several inequalities narrowed. Interventions are needed to improve child mental health while ensuring inequalities do not widen.

**WHAT IS ALREADY KNOWN ON THIS TOPIC**

- Declines in the mental health of adults and young people during the COVID-19 pandemic have been seen in most, but not all, studies on this topic. There is also some evidence that declines in mental health may have been greatest among younger people. However, the impact of the pandemic on inequalities in child mental health is not yet clear.

**WHAT THIS STUDY ADDS**

- Mental health among children declined overall during the pandemic, but this decline was greatest in traditionally advantaged groups such as children with employed parents or from higher-income households.

**HOW THIS STUDY MIGHT AFFECT RESEARCH, PRACTICE OR POLICY**

- Mental health during childhood is critical to health across the life course, by impacting on children’s engagement with education, and the establishment of positive health behaviours and relationships. Interventions that can improve the mental health of children across all groups are needed to address the impacts of the pandemic while maintaining narrower inequalities.

## Introduction

Childhood is a crucial life stage including important physical, socioemotional and cognitive developments. Social determinants of health experienced at this age have lasting consequences.^1^ Inequalities in material deprivation, housing and neighbourhood conditions, and access to quality childcare, education and health services lead to inequalities in health, which then reinforce socioeconomic disadvantage in a feedback loop.^2^ In the UK, children who have grown up in poverty are over three times as likely to experience mental health problems by age 14 years than those who have never experienced poverty.^3^ In 2020/2021, COVID-19 mitigation measures triggered an upheaval in children’s social environments, including the closure and dramatic changes to the delivery of childcare centres and schools, alongside a worsening economic outlook more broadly.^4^ These stressors are expected to affect child mental health in ways that long outlast the pandemic,^5^ especially among vulnerable groups.

Charities in the UK and other countries reported large increases in contacts from children and young people regarding their mental health linked to the pandemic.^6^ Child and adolescent mental health service data from Ireland similarly suggests that, while referrals initially dropped in spring 2020 they later rose compared with previous years, with an approximate doubling in urgent referrals in autumn 2020 compared with autumn 2019.^7^ Furthermore, empirical studies have suggested that mental health among young people worsened,^8–10^ and that younger age groups including primary-age children may have been worst affected.^9 11^ Studies in children aged under 11 years conducted during initial lockdowns revealed increases in emotional symptoms and attentional and conduct problems over the course of the pandemic,^11–13^ although to date most studies have focused on adolescents or older age ranges.

Much of the existing evidence base has relied on convenience samples, which tend to under-represent minority and disadvantaged groups. This is of particular concern because the mental health effects of the COVID-19 pandemic are unlikely to have been uniform across the population.^14^ Early in the pandemic teachers predicted that while some children might benefit from the easing of academic pressures, others might suffer from the added stresses of financial strain, overcrowded housing and lack of outdoor space.^15^ Studies in the UK and other countries have found that children experiencing socioeconomic disadvantages had worse mental health during the pandemic than those not experiencing these disadvantages.^9 10 16^ However, these studies have been unable to explore whether the disparity had changed compared with prepandemic inequalities.

We aimed to compare inequalities in mental health of similar aged primary school children both before and during the pandemic in the UK in a large, nationally representative sample. We use repeated survey data collected since 2009, with three additional time points across the pandemic, which allow population-level differences in mental health inequalities to be assessed beyond the initial months of lockdown.

## Method

### Study design and participants

Data were from Understanding Society: the UK Household Longitudinal Study (UKHLS).^17 18^ Since 2009, the study has annually surveyed individuals in households drawn from a cluster-stratified probability sample of postal addresses in the UK (referred to hereafter as the ‘main surveys’).^19^ In response to the pandemic, additional web-only surveys were conducted between April 2020 and March 2021 (‘COVID-19 surveys’).^20^ Response rates range between 65.9% and 83.8% for households in the main surveys,^21^ and between 38.0% and 66.5% for adults in the COVID-19 surveys (online supplemental file 1-1).

10.1136/jech-2022-220188.supp1 Supplementary data

All children in participating households and at eligible ages for mental health measures were included (online supplemental file 1-1). Members of the immigration and ethnicity boost sample, introduced in 2015, were excluded because enumeration weights for these children are not comparable to the rest of the sample. Information about child mental health was collected from parents of children aged 5 or 8 years between 2011 and 2019 in the main surveys. Additionally, in the July 2020, September 2020 and March 2021 COVID-19 surveys, parents were asked about the mental health of any children aged 5–11. Of the children (aged 5–11) eligible for Strengths and Difficulties Questionnaire (SDQ) measurement during the COVID-19 survey, 67% had also been eligible during the main (pre-COVID) survey (aged 5 or 8). Observations were weighted to account for non-response and survey design.^22^ Across both the main and COVID-19 surveys, a measure of child mental health was available for 85.9% of observations with valid survey weights, although missing SDQ data were higher among those from ethnic minority groups (excluding white ethnic minority groups), whose parents did not have degrees or were not employed and lived in low-income households. Non-response to covariates ranged from 0% to 11% (online supplemental file 1-2).

### Measurement

Child mental health was measured using the validated parental SDQ score which is sensitive to short-term interventions.^23 24^ The primary outcome was the ‘total difficulties score’. Higher scores represent greater psychosocial symptoms, which can include conduct or peer problems, hyperactivity inattention or emotional symptoms including anxiety and depression. Where both parents provided SDQ scores, the mother’s score was used in analysis. Additional sensitivity analysis used the father’s score in these cases (online supplemental file 1-3). In the main surveys, 97.8% of child SDQ scores were provided by mothers, whereas in the COVID-19 surveys this proportion was 82.6%. Sensitivity analyses used subscales designed to reflect internalising and externalising symptoms, and two binary SDQ score categorisations (>13 out of 40 indicating borderline-abnormal scores and >16 indicating abnormal scores).^25^

The impact of seven axes of inequality, which might modify the relationship between the COVID-19 pandemic and mental health, were investigated: sex; ethnicity (white, including white ethnic minorities, or other ethnic background); highest parental education (degree or lower); parental employment (at least one responding parent employed or not; with furloughed staff treated as employed); family structure (single or coupled parents); low equivalised net household income (<60% of the median or higher, based on the total weighted UKHLS sample average that year); local area deprivation (most deprived quintile within each country compared with all other quintiles). We performed sensitivity analyses comparing children whose parents have no formal educational qualifications to those whose parents are qualified to at least lower secondary (GCSE) level and comparing those living in the least deprived quintile of areas in their country to all others.

Net household income was adjusted for inflation and equivalised to take account of the number of residents.^26^ In the COVID-19 surveys, household income was measured using a truncated version of the main survey instrument.^27^ Local area deprivation was measured using the Index of Multiple Deprivation appropriate for each country.^28 29^ Each local area (Data Zones in Scotland, and Lower Super Output Areas in the rest of the UK) has been given a deprivation rank relative to other areas in that country. Ranks in different countries are not directly comparable and should be interpreted as indicating deprivation relative to other areas in the same UK country. Note that while area deprivation and household income are correlated, less than one-third of the sample who live in the most deprived quintile of areas also experience household poverty, and vice versa.

Ethnicity, sex and parental education were treated as time-invariant, with the most common response across all years used to replace any partially missing or inconsistent responses within each individual. Family structure and area deprivation, which were fairly stable over time, had missing values replaced by the closest previous or following response where possible, while still allowing for observed changes over time. Year and age at each observation were additional covariates.

### Statistical analyses

First, the sample is described before and during the COVID-19 pandemic. Second, mean SDQ scores for 5 and 8 year olds were calculated for the periods 2011–2013, 2014–2016 and 2017–2019 (collapsed to maximise power), stratified by each sociodemographic characteristic. A two-level generalised linear model was then used to estimate the mean SDQ score at ages 5 and 8 (the ages at which prepandemic SDQ measures were taken, to allow comparison between the main survey and COVID-19 survey samples) for 2020–2021 using the three relevant COVID-19 surveys, accounting for repeated observations. These models were weighted to adjust for survey design and non-response bias, by the inverse-probability of a child being included in any of the COVID-19 surveys^20^ (with or without weight sharing; online supplemental file 1-4.1). Each model used all observations with complete relevant data.

Third, to compare inequalities in mental health among similar-aged children both before and during the pandemic, we modelled the repeated survey data using two-level models to adjust for repeated observations of some children. A separate model was used for each sociodemographic variable, all adjusted for age, year and including interactions between the sociodemographic variables and age and year. Sex was also included as a covariate in the models for the other sociodemographic variables. A period variable indicated whether measures were taken before or after the pandemic onset. The parameter of interest was the interaction between this period variable and the sociodemographic variable. Data were weighted for sample attrition and survey design at the observation level (with no weighting at the child level). We assessed the performance of the weights, first, by comparing the analytical sample to those who responded to the earliest included survey (2011–2012), which has the lowest attrition. In general, the distributions of key characteristics were broadly similar, confirming that the sample remains representative (online supplemental file 1-5). Second, we examined sensitivity of results using four alternative methods to partition weights between the child level and observation level^30 31^ (online supplemental file 1-4.2), which all produced broadly similar results. Analyses were performed in Stata V.16.1.

## Results

The eligible sample consisted of 16 361 observations from 9272 children. Of these children, 1372 (14.8%) were observed both before and during the COVID-19 pandemic, 7226 (77.9%) were only measured prior, and 674 (7.3%) only measured during the pandemic. Table 1 describes the distribution of characteristics in the eligible sample, at the person-year level, before and after weighting for survey design and non-response. The unweighted distribution of characteristics at the person level are presented in online supplemental file 1-5. After weighting, the mean age was 7.0 years, 49.5% of observations were from female children, 17.4% were from ethnic minority groups (excluding white minority groups), 17.8% were from children living from poverty. Analysis samples were based on 7999 (86.3%) children in 14 018 (85.7%) observations with valid SDQ scores, age and sex (although each analysis additionally excludes children with missing data on the relevant sociodemographic variable, online supplemental file 1-2). Comparing the analytical sample to those responding in 2011–2012 (where attrition will have been lowest), reveals a lower proportion of children with unemployed parents that is not completely corrected for by weighting, although this is apparent in our analytical sample both before and during the pandemic (online supplemental file 1-5).

**Table 1:** Description of the sample at a person-year level before and after weighting, overall and stratified into those measured before and those measured during the COVID-19 pandemic

Characteristic	2011–2012 weighted sample %	Before the COVID-19 pandemic*		During the COVID-19 pandemic†		Total	
		Unweighted person-years (%)	Weighted person-years %	Unweighted person-years (%)	Weighted person-years %	Unweighted person-years (%)	Weighted person-years %
Sex							
Male	52.9	6101 (51.4)	50.9	2331 (52.5)	49.6	8432 (51.7)	50.5
Female	47.1	5766 (48.6)	49.1	2113 (47.6)	50.4	7879 (48.3)	49.5
Ethnicity							
White	83.5	8336 (71.4)	82.5	3306 (74.0)	82.6	11 642 (72.1)	82.6
Asian	6.2	1567 (13.4)	6.3	418 (9.4)	4.5	1985 (12.3)	5.7
Black	3.5	553 (4.7)	2.9	98 (2.2)	1.2	651 (4.0)	2.3
Mixed	6.0	1137 (9.7)	7.7	620 (13.9)	11.3	1757 (10.9)	8.8
Other	0.8	84 (0.7)	0.6	28 (0.6)	0.5	112 (0.7)	0.6
Lone parent	23.8	2306 (19.6)	22.1	527 (11.7)	17.8	2833 (17.4)	20.7
Highest parent education							
Degree	48.0	5773 (52.1)	51.7	2601 (61.1)	53.5	8374 (54.6)	52.3
Upper secondary education (A-level)	9.0	1067 (9.6)	9.3	443 (10.4)	9.6	1510 (9.9)	9.4
Lower secondary education (GCSE)	30.5	2743 (24.8)	27.7	730 (17.2)	27.6	3473 (22.7)	27.7
None	12.5	1493 (13.5)	11.3	481 (11.3)	9.4	1974 (12.9)	10.7
No parent employed	19.2	1819 (16.2)	15.9	491 (10.9)	14.9	2310 (14.7)	15.6
Low-income household	15.3	1695 (16.7)	15.9	850 (18.9)	21.7	2545 (17.4)	17.8
Resident in high deprivation area	22.1	2884 (24.4)	22.0	669 (15.0)	16.5	3553 (21.8)	20.2
Mean age (SD)	6.4 (1.4)	6.5 (1.5)	6.5 (1.4)	8.2 (1.9)	8.2 (1.7)	7.0 (1.8)	7.0 (1.7)
Mean SDQ score (SD)	8.6 (5.7)	8.5 (5.9)	8.7 (5.9)	8.8 (6.2)	9.6 (6.6)	8.6 (6.0)	9.0 (6.2)
Total N	1751	11 868	10 680‡	4493	4073‡	16 361	15 114‡

*Understanding Society surveys between 2011 and 2019.

†Understanding Society COVID-19 surveys in July 2020, September 2020, March 2021.

‡Person-years contributing to the weighted proportions.

GCSE, General Certificate of Secondary Education; SD, Standard deviation; SDQ, Strengths and Difficulties Questionnaire.

**Figure 1:**
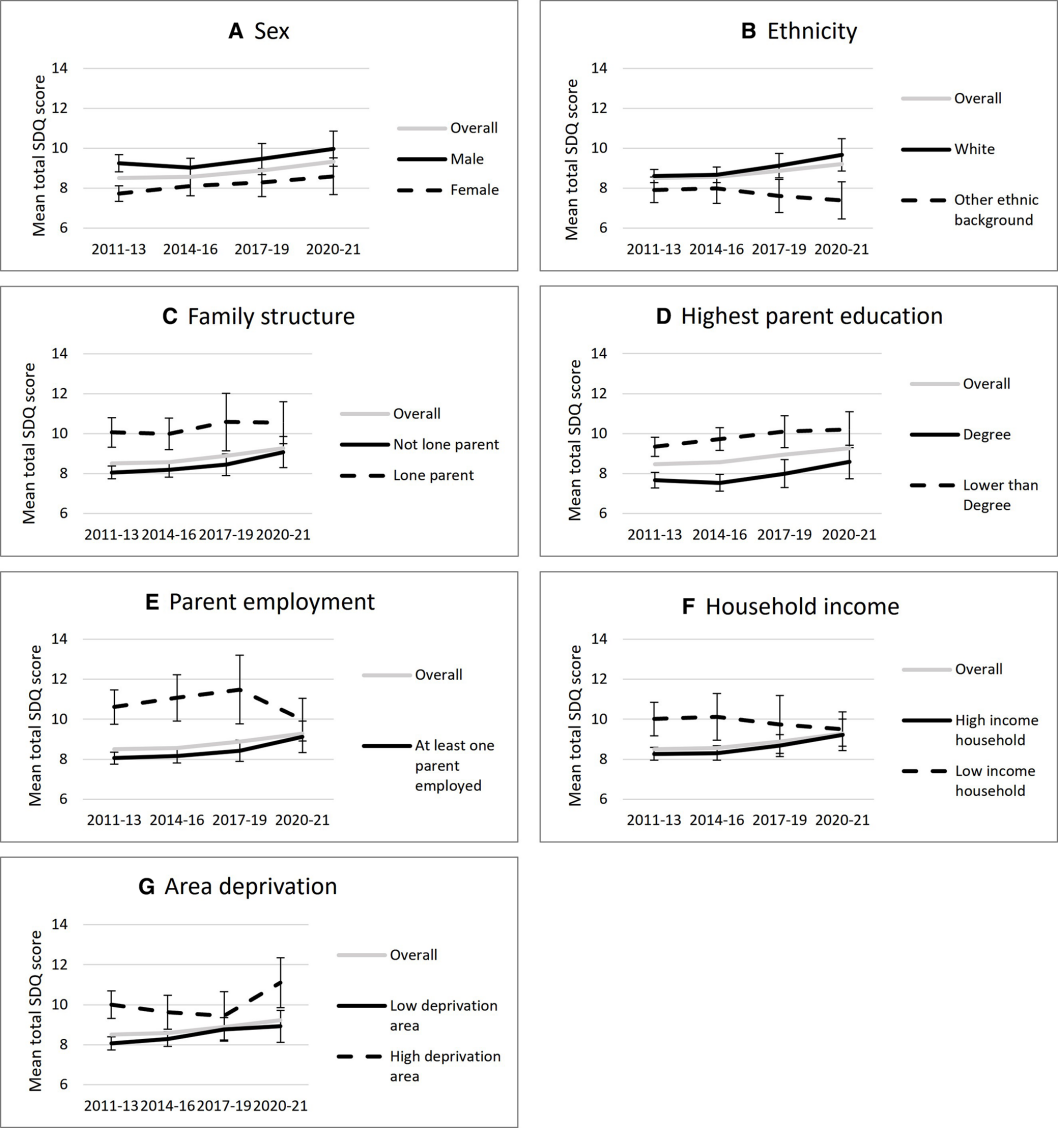
Trends in the total Strengths and Difficulties Questionnaire (SDQ) score, representing severity of mental health symptoms, among 5 year olds in the UK between 2011 and 2021. SDQ scores are presented stratified by seven measures of sociodemographic circumstance. (A) Sex (male or female) (B) ethnicity (white or not white) (C) family structure (single or coupled parenting) (D) highest parent education (degree or lower) (E) parent employment (at least one parent employed or no parent employed) (F) net household income (less than 60% of the median that year or higher) (G) area deprivation (resident in the 20% most deprived areas in that country or not). The vertical bars show the 95% CIs. Each graph includes all participants with an SDQ score and complete data on the sociodemographic variable in question, weighted for survey design and non-response.

Overall, mental health among this population had deteriorated over time. For example, the average SDQ total difficulties score of 5 year olds had risen from 8.32 out of 40 (95% CI 7.97 to 8.67) in 2011–2012 to 8.89 (95% CI 8.18 to 9.60) in 2018–2019, with higher scores representing worse mental health. During the pandemic, the average score rose further to 9.28 (95% CI 8.50 to 10.07) (figure 1). After adjustment for the linear time trend, age, and sex, the COVID-19 pandemic period was associated with a 0.17 point increase in SDQ scores (95% CI −0.22 to +0.55).

A larger rise in average SDQ score was often seen in typically advantaged groups, including children not living in poverty, children whose parents were educated to at least degree level, were employed or were parenting in a couple. In contrast, more disadvantaged groups, who tended to have lower mental health at baseline, experienced smaller declines in mental health during the COVID-19 pandemic. Consequently, several inequalities in child mental health narrowed during the pandemic, but this was driven by an overall decline in mental health. This pattern was less pronounced for inequalities by sex and area deprivation, which were maintained during the pandemic. White children (including white minority groups) had poorer baseline mental health than children from other ethnic backgrounds and experienced a larger decline in mental health during the COVID-19 pandemic, leading to a widening of this inequality. Figure 1 plots the average SDQ scores among 5-year-old children between 2011 and 2021, stratified by different sociodemographic variables. The same patterns were also found when SDQ scores among 8 year olds were plotted (online supplemental file 1-6).

**Figure 2:**
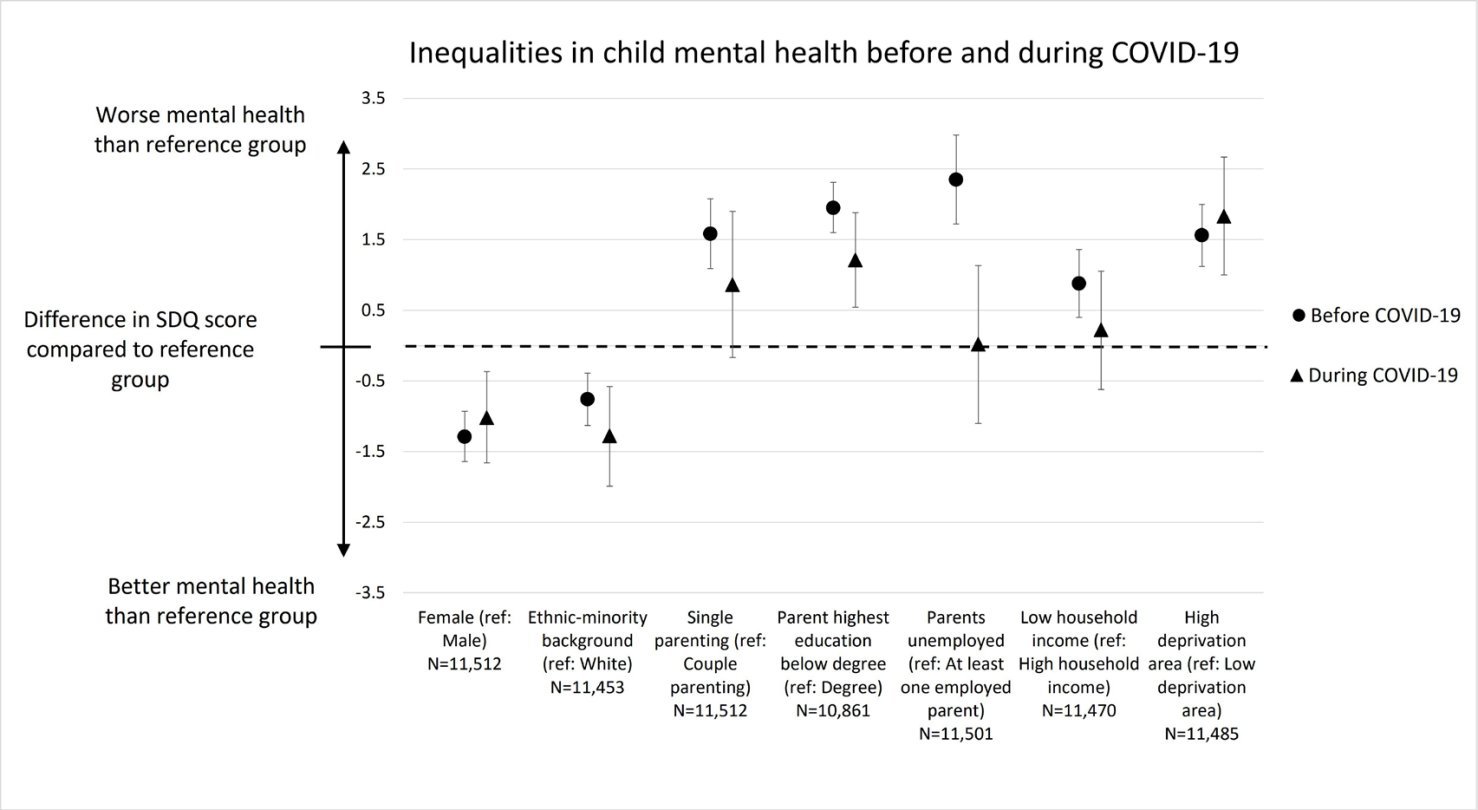
Differences in total Strengths and Difficulties Questionnaire (SDQ) score, representing severity of mental health symptoms, between different groups of the population compared with the reference groups, among children (aged 5–11 years) in the UK. Values are adjusted for child age, sex, year and the interactions between the relevant sociodemographic variable with age and year. The differences in SDQ score are plotted before the COVID-19 pandemic (circles) and during the COVID-19 (triangles) pandemic. The dotted line marks no difference in SDQ score. Points above this line indicate that this subgroup has worse mental health than the reference group, whereas points below the dotted line indicate the sub-group has better mental health than the reference. The vertical bars indicate the 95% CIs.

These patterns were also apparent in the multilevel models estimating the interactive effect of the pandemic and different sociodemographic characteristics on mental health, adjusted for child age, sex and year (figure 2). With the exceptions of sex, area deprivation and ethnicity, all other inequalities studied reduced during the COVID-19 pandemic. For example, the largest inequality in mental health measured before the pandemic was related to parent employment, where children with unemployed parents had SDQ scores on average 2.35 (95% CI 1.72 to 2.98) points higher (indicating poorer mental health) than children with employed parents. During the pandemic, this inequality had attenuated to only 0.02 points (95% CI −1.10 to +1.13).

The multilevel models again revealed a widening of the mental health inequality related to ethnicity, and that the poorer mental health experience of male children compared with female, and children living in the most deprived quintile of areas compared with all other areas, was maintained during the pandemic. When instead the experience of children living in the most affluent quintile of areas was compared with all other areas, a decrease in area deprivation inequalities in SDQ was apparent (online supplemental file 1-7.1), indicating that children living in this most affluent quintile of areas saw the largest declines in mental health. The narrowing of the inequality related to parent education was robust to changing the definition of lower parent education to compare children whose parents were educated to at least lower secondary (General Certificate of Secondary Education[GCSE]) level or not (rather than degree level or not; online supplemental file 1-7.2).

The patterns described were apparent for both internalising and externalising mental health symptoms (online supplemental file 1-8). Decreases in mental health inequalities relating to parent education, employment and household income were also replicated when SDQ scores were categorised into a binary outcome and compared using risk ratios. For example, before the COVID-19 pandemic, the risk of experiencing borderline-abnormal mental health (SDQ score>13) was 2.18 times higher among children with unemployed parents than children with employed parents (95% CI 1.88 to 2.52), but only 1.38 times higher during the pandemic (95% CI 1.00 to 1.90) (online supplemental file 1-9). Results were also robust to the use of alternative weighting strategies (online supplemental file 1-4.2), and to using SDQ scores provided by fathers or by mothers for the analysis in cases where responses from both parents are available (online supplemental file 1-3).

## Discussion

Mental health symptoms among UK children aged 5–11 years increased between 2011 and 2019, a trend that continued into the pandemic. Prior to the COVID-19 pandemic, disadvantaged groups generally had worse mental health than more advantaged groups. During the pandemic, many of these inequalities narrowed as the mental health of children in more advantaged groups saw greater deteriorations. This pattern was strongest when comparing children with unemployed to employed parents and was also apparent for inequalities related to family structure, parent education and household income. In contrast, concerningly inequalities related to ethnicity widened, with white children (including white British and white ethnic minority groups) experiencing worse mental health than those from other ethnic backgrounds throughout, and a larger decline in mental health during the pandemic. Furthermore, disadvantages related to male sex and neighbourhood deprivation were maintained.

### Comparison to other studies

This study benefited from a large, population-representative sample, allowing a range of inequalities to be examined. The finding that advantaged children experienced greater increases in mental health symptoms during the COVID-19 pandemic has been replicated in other smaller studies. One study of 4–16 year olds in spring 2020 found that although children living in low-income households displayed elevated conduct issues at baseline, it was the more advantaged comparison group that saw the greatest increases over the 4 months studied.^32^ The finding that white children experienced worse mental health prior to the pandemic is also consistent with the findings of a systematic review describing the prevalence of common mental health disorders among children from different ethnic backgrounds,^33^ although not all studies agree,^34^ and the reasons for these differences are unclear.^33^ A survey of 5–22 year olds found, similar to our study, that while average mental health declined between 2017 and July 2020, the decline was greater in white children than children from black and ethnic minority backgrounds, and that white children started from a poorer level of mental health at baseline.^35^ Continuing to investigate ethnic differences in mental health using samples sizes large enough to explore the experiences of different groups will be important for understanding the significance of these trends over time. The survey of 5–22 year olds did not find evidence for inequalities in mental health related to area deprivation, however, area deprivation is widely known to be associated with mental health,^36^ and our study indicates that children in more deprived areas experienced worse mental health both before and during the pandemic.

There are additionally some indications that similar patterns may have been observed outside the UK. A cross-sectional study of 2–18 year olds in Canada found that greater economic concerns were associated with parent-reported or self-reported improvements in anxiety and attention compared with before the pandemic.^37^ Additionally, a study of emergency department presentations between 2018 and 2020 in Victoria, Australia found that the proportion of children from more socially advantaged areas presenting for self-harm or developmental and behavioural disorders increased slightly during the pandemic compared with the preceding years.^38^

In contrast, a study of 10–11 year olds in Wales found that absolute inequalities in emotional problems increased between two time points in 2019 and 2021, with no change in behavioural difficulties.^8^ The difference to our results may partly reflect the older age distribution of children or differing geographic context. Further studies found that socioeconomic inequalities in mental health among young people were maintained during the pandemic, however, most relied on smaller sample sizes, were not nationally representative, and examined older age ranges.^39–41^ Our study uses measurements covering the first year of the pandemic and compares these to measurements over the previous decade.

### Limitations

An important limitation of our study is that survey and item non-response may have introduced selection bias into our analysis. Adult participation rates in UKHLS (including both parents and non-parents) were between 38.0%–66.5% and were more variable in the COVID-19 surveys than the main surveys (online supplemental file 1-1). Inverse probability weights were used to adjust for predictable non-response, however, the proportion of children living in deprived areas was lower than expected during the pandemic, a pattern that was not fully corrected by use of survey weights. Furthermore, children with missing SDQ scores were more likely to be from disadvantaged groups, although weighting partially corrected for this issue for some measures, such as ethnicity (online supplemental file 1-2). If the parents of disadvantaged children who were experiencing poor mental health were less likely to fully respond to the survey during the pandemic, our estimates of inequality during the pandemic could be underestimated. Thus, selection bias could explain part of the narrowing of inequality described during the COVID-19 pandemic. Nevertheless, our results were robust to multiple weighting strategies, which we developed to account for attrition and selection bias (online supplemental file 1-4) and the impact of missing item data was minimised by carrying forward responses for stable variables.

A second limitation is that child SDQ scores were calculated using parent-reported symptoms. The SDQ score is most robust as a measure of mental health when reported from multiple sources (by parents and teachers).^23^ More time spent with children during lockdown and pressures of home-schooling might impact how parents report symptoms, which may have varied between comparison groups.

Third, small sample sizes necessitated the aggregation of minority ethnicities, which may mask important differences between groups. For example, previous research has suggested that among South Asian ethnicities, children from Indian ethnic backgrounds tend to have better mental health than white British children, whereas the mental health of children from Pakistani and Bangladeshi ethnic backgrounds tend to be similar to the white British group.^33^ Similarly, using binary measures for each measure of socioeconomic circumstance may obscure the gradients of disadvantage experienced within each category. Nevertheless, similar results were found when different thresholds were selected to define low education or area deprivation (online supplemental file 1-7).

Fourth, the shorter instrument used to measure household income in the COVID-19 surveys has been shown to generate greater variance in measurement error and lead to under-reporting of income compared with the main survey instrument,^27^ however, household income was explored relative to other houses in the same survey wave so direct comparisons across measurement approaches were not made.

### Meaning and implications

Our study provides evidence that trends in child mental health have continued to worsen during the pandemic. Unexpectedly, in many cases children from traditionally advantaged groups saw larger declines than children from disadvantaged groups, that is, child mental health has become more equal but at a worse overall level. The pattern is contrary to predictions from some child health experts that the financial and emotional strain of lockdowns would fall hardest on children with parents in unstable employment, living in overcrowded housing, with less access to outdoor space and educational resources.^14^ Between March 2020 and March 2021, children experienced significant disruption to social activity and education, with schools operating largely remotely except for a brief period in autumn 2020. We speculate that social isolation and reduced access to services during the COVID-19 pandemic brought the experiences of traditionally advantaged groups closer to those already faced by children from disadvantaged backgrounds, and/or that emergency income support measures during the pandemic may have eased the economic burden for disadvantaged families.

The difference in prepandemic and during pandemic mental health inequalities was greatest comparing children with employed and unemployed parents, which may result from changes in the composition of unemployed groups during the pandemic. Furthermore, parents experienced increased childcare responsibilities during the pandemic while schools were closed. This strain, which has been linked to parent distress levels,^42^ may plausibly have been greatest for families with employed parents who needed to balance childcare against their paid work. Moreover, the intense pressures and increased risk of SARS-CoV-2 infection faced by essential workers in this period may have placed further strain on some families with employed parents. For example, studies in Brazil and Bangladesh have shown that children whose parents worked in essential roles, and were unable to work from home, experienced worse mental health during the pandemic.^43 44^ We speculate that these excess pressures faced by some working parents, who were required to balance childcare and paid work during the pandemic, may have contributed to the poorer mental health of children with employed parents during the pandemic compared with before. Further research exploring the factors driving changes in child mental health inequalities is needed to assess these hypotheses.

These findings show the negative consequences of social disadvantage across the socioeconomic gradient. Furthermore, while many socioeconomic inequalities in mental health narrowed during the pandemic, inequalities related to area deprivation were maintained. Area deprivation captures disadvantage at a community level, including access to services, crime, and the quality of the local environment. Although correlated with individual-level socioeconomic circumstance, in Scotland, for example, less than half of income-deprived individuals live in the most deprived areas.^45^ The maintained worse mental health of children living in deprived neighbourhoods emphasises the importance of service provision including childcare, healthcare and safe places to play. These structures create sources of resilience for families and routines for children, which parents highlighted as key to child well-being during the COVID-19 pandemic.^46^

These results have implications for ensuring that inequalities in children’s mental health do not rewiden now that COVID-19 restrictions have lifted in many countries. Understanding the long-term consequences of the pandemic for child mental health, and how it intersects with different domains of disadvantage, is important for planning mental health service delivery and intervening on the causes of declining child mental health. Our findings support calls from academics, child health organisations and psychologists for a renewed global focus on child mental health in research and service planning,^12 47^ with a Health in All Policies approach.

## Conclusion

In conclusion, our findings show that some inequalities in the mental health of 5 and 8 year olds have reduced during the COVID-19 pandemic, but that this occurred in the context of an overall decline in their mental health. The implications of this decline are particularly important because poor mental health as a child has ramifications across the life course, including effects on children’s ability to engage in education.^2^ Interventions are urgently needed to improve child mental health across all groups, while seeking to maintain the narrower inequalities observed during the first year of the pandemic via upstream policies to reduce socioeconomic disadvantage.

## Data Availability

Data are available in a public, open access repository. The UKHLS main survey and COVID survey data are publicly available from the UK Data Service (study numbers 6614 and 8644) at: https://beta.ukdataservice.ac.uk/datacatalogue/series/series?id=2000053. Use of individual-level data is governed by an UK Data Service End User Licence. The use of area-level data is provided under a UK Data Service special license (SN 7248). The STATA code is available on request to the corresponding author.

## References

[1] Graham H , Power C . Childhood disadvantage and adult health: a Lifecourse framework. London: Health Development Agency, 2004. Available: https://citeseerx.ist.psu.edu/viewdoc/download?doi=10.1.1.486.4643&rep=rep1&type=pdf 10.1111/j.1365-2214.2004.00457.x 15527477

[2] Pearce A , Dundas R , Whitehead M , et al . Pathways to inequalities in child health. Arch Dis Child 2019;104:998–1003. 10.1136/archdischild-2018-314808 30798258 PMC6889761

[3] Lai ETC , Wickham S , Law C , et al . Poverty Dynamics and health in late childhood in the UK: evidence from the millennium cohort study. Arch Dis Child 2019;104:1049–55. 10.1136/archdischild-2018-316702 31186294 PMC6837248

[4] Sinha I , Bennett D , Taylor-Robinson DC . Children are being sidelined by COVID-19. BMJ 2020;369:m2061. 10.1136/bmj.m2061 32461203

[5] Hefferon C , Taylor C , Bennett D , et al . Priorities for the child public health response to the COVID-19 pandemic recovery in England. Arch Dis Child 2021;106:533–8. 10.1136/archdischild-2020-320214 33298551

[6] Organisation for Economic Co-operation and Development (OECD) . Supporting young people’s mental health through the COVID-19 crisis. 2021. Available: https://www.oecd.org/coronavirus/policy-responses/supporting-young-people-s-mental-health-through-the-covid-19-crisis-84e143e5/

[7] McNicholas F , Kelleher I , Hedderman E , et al . Referral patterns for specialist child and adolescent mental health services in the Republic of Ireland during the COVID-19 pandemic compared with 2019 and 2018. BJPsych Open 2021;7:e91. 10.1192/bjo.2021.48 33938419 PMC8111180

[8] Moore G , Anthony R , Angel L , et al . Mental health and life satisfaction among 10–11-year-olds in Wales, before and one year after onset of the COVID-19 pandemic. BMC Public Health 2022;22:379. 10.1186/s12889-022-12752-6 35193528 PMC8863505

[9] Ravens-Sieberer U , Kaman A , Erhart M , et al . Impact of the covid-19 pandemic on quality of life and mental health in children and adolescents in germany. Eur Child Adolesc Psychiatry 2022;31:879–89. 10.1007/s00787-021-01726-5 33492480 PMC7829493

[10] Li W , Wang Z , Wang G , et al . Socioeconomic inequality in child mental health during the COVID-19 pandemic: first evidence from China. J Affect Disord 2021;287:8–14. 10.1016/j.jad.2021.03.009 33761325 PMC9754677

[11] Creswell C , Shum A , Pearcey S , et al . Young people’s mental health during the COVID-19 pandemic. Lancet Child Adolesc Health 2021;5:535–7. 10.1016/S2352-4642(21)00177-2 34174991 PMC9765398

[12] Watson M et al . COVID-19 early years resilience and impact survey (CEYRIS). background report. Edinburgh Public Health Scotland; 2020. Available: https://publichealthscotland.scot/media/3103/background-report_ceyris.pdf

[13] Newlove-Delgado T , McManus S , Sadler K , et al . Child mental health in England before and during the COVID-19 Lockdown. Lancet Psychiatry 2021;8:353–4. 10.1016/S2215-0366(20)30570-8 33444548 PMC8824303

[14] Millar R et al . Considering the evidence of the impacts of Lockdown on the mental health and wellbeing of children and young people within the context of the individual, the family, and education. Mental Health Foundation; 2020. Available: https://www.mentalhealth.org.uk/sites/default/files/2022-08/MHF-Scotland-Impacts-of-lockdown.pdf

[15] YoungMinds . Impact of COVID-19 on children and young people’s mental health: results of survey with teachers and school staff. 2020. Available: https://www.youngminds.org.uk/media/0ajhy5kl/youngminds-survey-with-school-staff.pdf

[16] Pearcey S . Report 04: changes in children and young people’s emotional and behavioural difficulties through Lockdown; 2020. Available: https://emergingminds.org.uk/wp-content/uploads/2020/06/CoSPACE-Report-4-June-2020.pdf

[17] Institute for Social and Economic Research University of Essex, Understanding Society . Waves 1-11, 2009-2020 and Harmonised BHPS: waves 1-18, 1991-2009. In: UK Data Service. 2022. 10.5255/UKDA-SN-6614-16

[18] Institute for Social and Economic Research University of Essex, Understanding Society . COVID-19 study, 2020-2021. In: UK Data Service. 2021. 10.5255/UKDA-SN-8644-11

[19] Institute for Social and Economic Research . Understanding society: waves 1-11, 2009-2020 and Harmonised BHPS: waves 1-18, 1991-2009, user guide. Colchester University of Essex; 2021. Available: https://www.understandingsociety.ac.uk/sites/default/files/downloads/documentation/mainstage/user-guides/mainstage-user-guide.pdf [Accessed 30 Oct 2021].

[20] Institute for Social and Economic Research . Understanding Society COVID-19 User Guide. Version 10.0. Colchester: University of Essex, 2021. Available: https://www.understandingsociety.ac.uk/sites/default/files/downloads/documentation/covid-19/user-guides/covid-19-user-guide.pdf [accessed Oct 2021].

[21] Institute for Social and Economic Research . Response tables. Colchester University of Essex. n.d. Available: https://www.understandingsociety.ac.uk/sites/default/files/downloads/documentation/user-guides/mainstage/responsetables.pdf

[22] Kaminska O , Lynn P . Weighting and sample representation: frequently asked questions. Colchester Institute for Social and Economic Research, University of Essex; 2019. Available: https://www.understandingsociety.ac.uk/sites/default/files/downloads/documentation/user-guides/mainstage/weighting_faqs.pdf

[23] Early Intervention Foundation . Strengths and difficulties questionnaire (SDQ). 2020. Available: https://www.eif.org.uk/files/resources/measure-report-child-sdq.pdf

[24] Stone LL , Otten R , Engels RCME , et al . Psychometric properties of the parent and teacher versions of the strengths and difficulties questionnaire for 4- to 12-year-olds: A review. Clin Child Fam Psychol Rev 2010;13:254–74. 10.1007/s10567-010-0071-2 20589428 PMC2919684

[25] Effective Health Care Alliance Programme . Scoring the strengths & difficulties questionnaire for age 4-17. 2014. Available: https://www.ehcap.co.uk/content/sites/ehcap/uploads/NewsDocuments/236/SDQEnglishUK4-17scoring-1.PDF

[26] Organisation for Economic Co-operation and Development (OECD) . What are equivalence scales? OECD project on income distribution and poverty. n.d. Available: https://www.oecd.org/economy/growth/OECD-Note-EquivalenceScales.pdf

[27] Crossley T , Fisher P , Hussein O . Assessing earnings and income data from a short web survey, understanding society working paper 2022-01. Colchester University of Essex; 2022. Available: https://www.understandingsociety.ac.uk/sites/default/files/downloads/working-papers/2022-01.pdf

[28] Institute for Social and Economic Research University of Essex, Understanding Society . Waves 1-11, 2009-2020 and Harmonised BHPS: waves 1-18, 1991-2009: special licence access, census 2001 lower layer super output areas. In: UK Data Service. 2021. 10.5255/UKDA-SN-6670-13

[29] Institute for Social and Economic Research University of Essex . Understanding society: COVID-19 study, 2020-2021: special licence access, census 2011 lower layer super output areas. In: UK Data Service. 2021. 10.5255/UKDA-SN-8663-7

[30] Rabe-Hesketh S , Skrondal A . Multilevel Modelling of complex survey data. Journal of the Royal Statistical Society Series A 2006;169:805–27. 10.1111/j.1467-985X.2006.00426.x

[31] Carle AC . Fitting Multilevel models in complex survey data with design weights: recommendations. BMC Med Res Methodol 2009;9:49. 10.1186/1471-2288-9-49 19602263 PMC2717116

[32] Raw JAL , Waite P , Pearcey S , et al . Examining changes in parent-reported child and adolescent mental health throughout the UK’s first COVID-19 national Lockdown. J Child Psychol Psychiatry 2021;62:1391–401. 10.1111/jcpp.13490 34327726 PMC8447308

[33] Goodman A , Patel V , Leon DA . Child mental health differences amongst ethnic groups in Britain: a systematic review. BMC Public Health 2008;8:258. 10.1186/1471-2458-8-258 18655701 PMC2515844

[34] Zilanawala A , Sacker A , Nazroo J , et al . Ethnic differences in children’s Socioemotional difficulties: findings from the millennium cohort study. Soc Sci Med 2015;134:95–106. 10.1016/j.socscimed.2015.04.012 25931288

[35] Vizard T et al . Mental health of children and young people in England. NHS; 2020. Available: https://files.digital.nhs.uk/AF/AECD6B/mhcyp_2020_rep_v2.pdf

[36] Marryat L , Thompson L , Minnis H , et al . Primary schools and the amplification of social differences in child mental health: a population-based cohort study. J Epidemiol Community Health 2018;72:27–33. 10.1136/jech-2017-208995 29056594 PMC5753027

[37] Cost KT , Crosbie J , Anagnostou E , et al . Mostly worse, occasionally better: impact of COVID-19 pandemic on the mental health of Canadian children and adolescents. Eur Child Adolesc Psychiatry 2022;31:671–84. 10.1007/s00787-021-01744-3 33638005 PMC7909377

[38] Hiscock H , Chu W , O’Reilly G , et al . Association between COVID-19 restrictions and emergency Department presentations for Paediatric mental health in Victoria, Australia. Aust Health Rev 2022;46:529–36. 10.1071/AH22015 35787299

[39] Patrick SW , Henkhaus LE , Zickafoose JS , et al . Well-being of parents and children during the COVID-19 pandemic: A national survey. Pediatrics 2020;146:e2020016824. 10.1542/peds.2020-016824 32709738

[40] James M , Marchant E , Defeyter MA , et al . Impact of school closures on the health and well-being of primary school children in Wales UK: a routine data linkage study using the HAPPEN survey (2018–2020). BMJ Open 2021;11:e051574. 10.1136/bmjopen-2021-051574 PMC8503919 34625414

[41] Bignardi G , Dalmaijer ES , Anwyl-Irvine AL , et al . Longitudinal increases in childhood depression symptoms during the COVID-19 Lockdown. Arch Dis Child 2021;106:791–7. 10.1136/archdischild-2020-320372 PMC7733224 33298552

[42] Green MJ , Craig P , Demou E , et al . Understanding inequalities in mental health by family structure during COVID-19 lockdowns: evidence from the UK household longitudinal study. Epidemiology [Preprint] 2022. 10.1101/2022.10.27.22281616 PMC10242239 37280641

[43] Garcia de Avila M , Hamamoto Filho P , Jacob F , et al . Children’s anxiety and factors related to the COVID-19 pandemic: an exploratory study using the children’s anxiety questionnaire and the numerical rating scale. IJERPH 2020;17:5757. 10.3390/ijerph17165757 32784898 PMC7459447

[44] Yeasmin S , Banik R , Hossain S , et al . Impact of COVID-19 pandemic on the mental health of children in Bangladesh: A cross-sectional study. Child Youth Serv Rev 2020;117:105277. 10.1016/j.childyouth.2020.105277 32834275 PMC7387938

[45] McCartney G , Hoggett R . How well does the Scottish index of multiple deprivation identify income and employment deprived individuals across the urban-rural spectrum and between local authorities? Public Health 2023;217:26–32. 10.1016/j.puhe.2023.01.009 36841036

[46] Chambers S et al . Parents' perceptions of children’s emotional well-being during spring 2020 COVID-19 restrictions: A qualitative study with parents of young children in England. Child: Care, Health and Development, 10.1111/cch.13034 PMC9349486 35839296

[47] The Early Childhood Development Action Network . A joint statement on early childhood development and COVID-19: a call for coordinated action to protect and support all young children and their Caregivers. 2020. Available: https://mcusercontent.com/8103bc6125ed66e0964ae244d/files/1919e510-55e7-4500-9473-188278e1a31d/CallToAction_04_10_2020_Noon.pdf

