# Supplementary material for: Inequalities in children’s mental health before and during the COVID-19 pandemic: findings from the UK Household Longitudinal Study
Source: J Epidemiol Community Health. 2023 Sep 25;77(12):762–9. doi: 10.1136/jech-2022-220188 (PMC10646900; doi:10.1136/jech-2022-220188)
Supplement: Supplementary data [file jech-2022-220188supp001.pdf]

Supplementary file 1

Contents

1. Participant flow diagram
2. Investigating missing item data
3. Exploring sensitivity to which parent provides the SDQ values during the COVID-19 pandemic
4. Analyses using alternative weighting mechanisms
   1. Trends in average SDQ scores stratified by each axis of inequality using an alternative weighting system for the COVID-survey scores
   2. Multi-level model results using four alternative weighting schemes
5. Description of the sample characteristics at child level and person-year level
6. Trends in cross-sectional average SDQ score among 8-year-olds
7. Exploring sensitivity to categorisations of area deprivation and higher education
   1. Alternative categorisations of area deprivation
   2. Alternative categorisations of higher education
8. Analysis of externalised and internalised child mental health symptoms
9. Analysis of proportion of children with abnormal SDQ scores

Supplementary file 1-1. Participant flow diagram

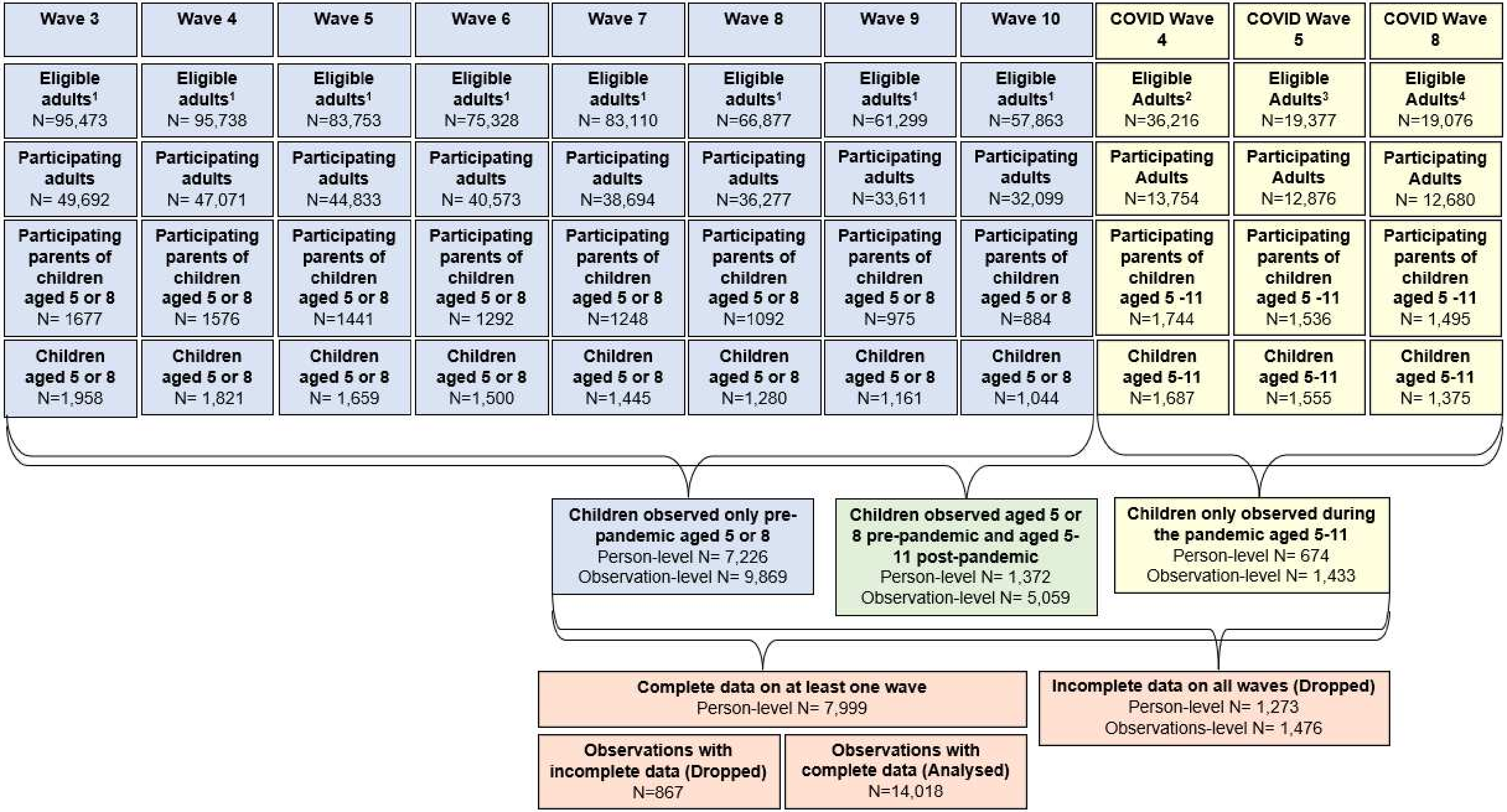

Figure S1 The source of the eligible participants and observations analysed from the UK Household Longitudinal Study

[1]Eligble adults in Main Survey waves 3-10 are from sampled households in the General Population sample and Ethnic Minority Boost sample of the UK Household Longitudinal Study

(UKHLS), and respondents of the British Household Panel Survey who chose to also join UKHLS.
[2]Adults issued the COVID Wave 4 survey are the members of households which participated in wave 8 or wave 9 of the main Understanding Society surveys.
[3]Adults issued the COVID Wave 5 survey are any participants who had given at least one partial interview in the COVID surveys during waves 1 to 4.
[4]Adults issued the COVID Wave 8 survey are any participants who had given at least one partial interview in the COVID surveys during waves 2 to 5.

Supplementary file 1-2. Investigating missing item data

Table S1 Proportion of children and observations with missing item data on each variable, from a sample of 9,272 children (16,361 observations) aged 5 or 8 and participating in the main surveys of the UK Longitudinal Household Study or aged 5-11 in the COVID-19 surveys.

**Characteristic**	**Number of children with at least one missing value (%)**	**Number of person-years with a missing value (%)**
SDQ score	2,062 (22.2)	2,306 (14.1)
Sex	37 (0.4)	50 (0.3)
Ethnicity	160 (1.7)	214 (1.3)
Family structure	89 (1.0)	112 (0.7)
Highest parent education	641 (6.9)	1,030 (6.3)
Parent employment	568 (6.1)	608 (3.7)
Household income	1,448 (15.6)	1,716 (10.5)
Area deprivation	26 (0.3)	45 (0.3)

Table S2 Comparing the distribution of characteristics in observations with a valid SDQ score, compared to those where an SDQ score could not be calculated. Differences in the distribution of characteristics between the observations without an SDQ score from the entire sample suggest that children with missing SDQ score observations may differ meaningfully from the rest of the sample, introducing selection bias. Weighting partially accounts for this issue in some cases. For example, the estimated proportion of observations without an SDQ score in each ethnic group is more similar to the weighted proportions in the entire sample after weighting than before.

**Characteristic**	Proportion of person-years with an SDQ score that fall in this group		Proportion of person-years with missing SDQ score that fall in this group		Total proportion of person-years	
	Unweighted	Weighted	Unweighted	Weighted	Unweighted	Weighted
**Sex**						
Male	51.7	50.5	51.7	50.1	51.7	50.5
Female	48.3	49.5	48.3	49.9	48.3	49.5
**Ethnicity**						
White	75.5	83.6	51.3	70.2	72.1	82.6
Asian	9.8	5.1	27.9	13.4	12.3	5.7
Black	3.3	1.9	8.9	7.1	4.0	2.3
Mixed	11.0	8.9	10.1	7.8	10.9	8.8
Other	0.5	0.5	1.8	1.5	0.7	0.6
**Lone parent**	17.2	20.3	19.0	25.4	17.4	20.7
**Highest parent education**						
Degree	55.9	53.2	47.1	41.9	54.6	52.3
Upper secondary (A-level)	9.8	9.4	10.2	8.8	9.9	9.4
Lower secondary (GCSE)	22.1	27.3	26.3	32.2	22.7	27.7
None	12.3	10.1	16.4	17.1	12.9	10.7
**No parent employed**	13.5	14.8	24.0	24.1	14.7	15.6
**Low-income household**	16.3	17.3	24.9	23.6	17.4	17.8
**Resident in high deprivation area**	19.9	19.4	33.3	30.0	21.8	20.2
**Mean age years (95% CI)**	7.0 (7.0-7.1)	7.1 (7.0-7.1)	6.7 (6.6-6.8)	6.8 (6.7-7.0)	7.0 (7.0-7.0)	7.0 (7.0-7.1)
**Total (N)**	14,055	13,706	2,306	1,269	16,361	15,114

Supplementary file 1-3. Exploring sensitivity to which parent provides the SDQ values during the COVID-19 pandemic

Table S3 Results of a mixed effects generalised linear model comparing total SDQ scores between different groups of children aged 5 and 8 in the UK Longitudinal Household Study main survey or 5-11 in the COVID-19 surveys, before and during the pandemic. The first two columns show results when SDQ scores provided by the mothers are used wherever both parents have provided an SDQ score for the child, whereas the last two columns show the results when the fathers response is used in these cases. Results are similar.

	**Using mother SDQ score where both parents have responded**		**Using father SDQ score where both parents have responded**	
**Characteristic**	**Difference in SDQ before the pandemic**	**Difference in SDQ during the pandemic**	**Difference in SDQ before the pandemic**	**Difference in SDQ during the pandemic**
Female (ref: Male)	-1.29 (-1.64 to -0.93)	-1.02 (-1.66 to -0.37)	-1.23 (-1.59 to -0.87)	-1.10 (-1.76 to -0.44)
Non-white ethnicity (ref: White)	-0.76 (-1.13 to -0.39)	-1.28 (-1.99 to -0.58)	-0.82 (-1.20 to -0.45)	-1.11 (-1.85 to -0.37)
Single parenting (ref: Couple parenting)	+1.58 (+1.09 to +2.08)	+0.86 (-0.17 to +1.90)	+1.63 (+1.14 to +2.12)	+0.67 (-0.37 to +1.71)
Parent highest education below degree (ref: Degree)	+1.95 (+1.60 to +2.31)	+1.21 (+0.54 to +1.88)	+1.97 (+1.61 to +2.32)	+0.97 (+0.28 to +1.66)
Parents unemployed (ref: At least one employed parent)	+2.35 (+1.72 to +2.98)	+0.02 (-1.10 to +1.13)	+2.45 (+1.83 to +3.08)	+0.05 (-1.07 to +1.18)
Low household income (ref: High household income)	+0.88 (+0.40 to +1.36)	+0.22 (-0.62 to +1.05)	+0.84 (+0.34 to +1.33)	-0.22 (-1.07 to +0.63)
High deprivation area (ref: Low deprivation area)	+1.56 (+1.12 to +2.00)	+1.83 (+1.00 to +2.67)	+1.61 (+1.18 to +2.05)	+1.46 (+0.64 to +2.28)

Supplementary file 1-4. Analyses using alternative weighting mechanisms

Supplementary file 1-4.1. Inverse probability weights were assigned to children based on the main survey design and non-response weights for their parents (with a preference for mothers) and whether or not the parent took part in the relevant COVID surveys. Analysis of trends in cross-sectional mean SDQ scores was repeated using an alternative child-level weight to calculate the estimated mean 2020-2021 SDQ scores. The weight used in the main analysis uses a weight-sharing mechanism, distributing weights to parents who were in the COVID survey, but not the preceding main waves, based on the weights of parents with similar characteristics who were given a weight in the main survey. The weight used in this supplementary analysis does not employ the weight sharing mechanism, so only parents appearing in both the preceding main survey and the COVID survey are given a weight. Results are similar.

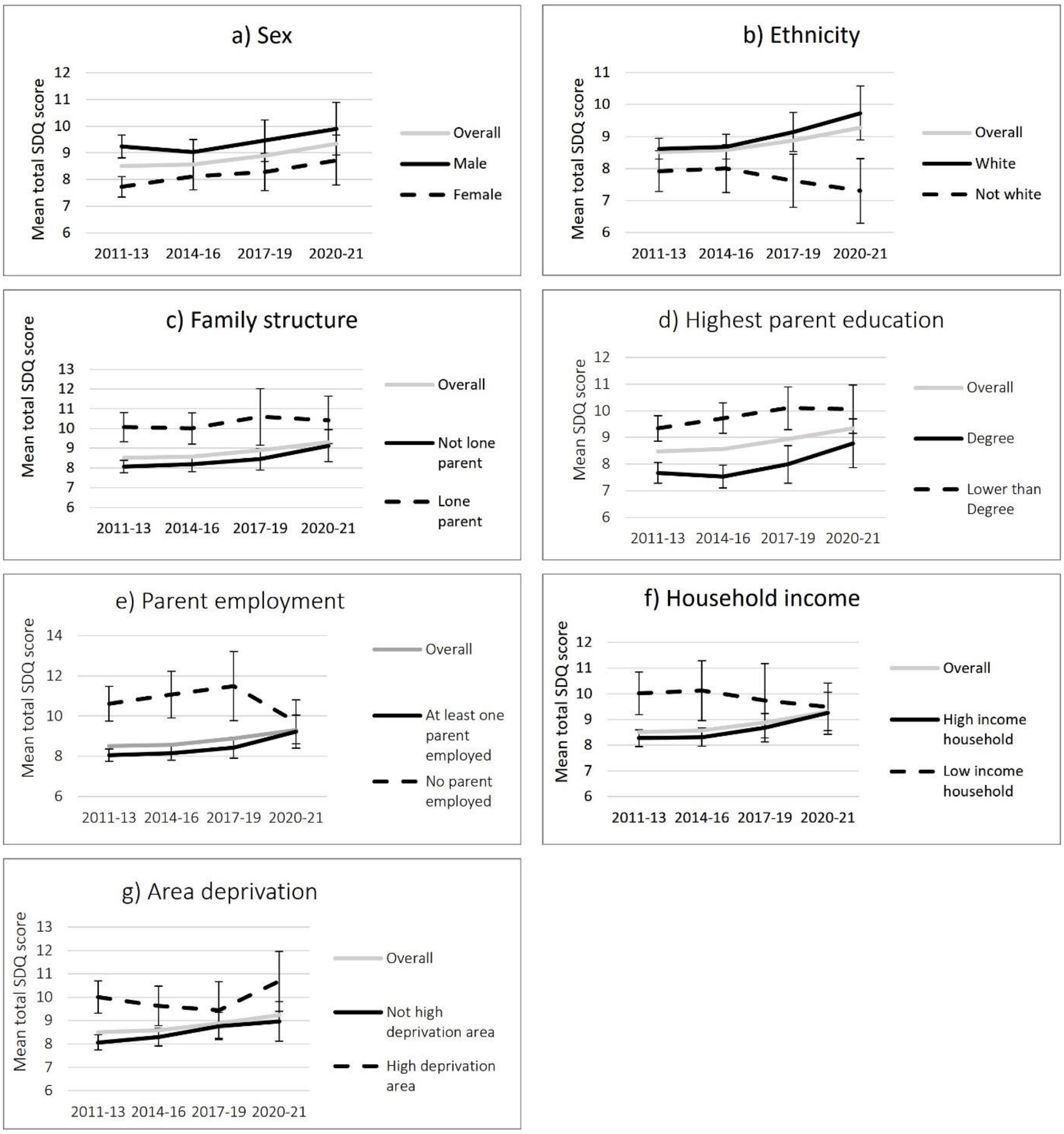

Figure S2 Trends in the total SDQ score, representing severity of mental health symptoms, amongst 5 year olds in the UK between 2011 and 2021. SDQ scores are presented stratified by seven measures of inequality. a) sex (male or female) b) ethnicity (white or not white) c) family structure (single or coupled parenting) d) highest

parent education (degree or lower) e) parent employment (at least one parent employed or no parent employed) f) household income (less than 60% of the median that year or higher) g) area deprivation (resident in the 20% most deprived areas in that country or not). The vertical bars show the 95% confidence intervals. Each graph includes all participants with a SDQ score and complete data on the inequality in question, weighted for survey design and non-response.

Supplementary file 1-4.2. Four alternative weighting mechanisms were also used in the mixed effects generalised linear model to explore the impact of the pandemic on mental health inequalities. The main analysis used weights provided for parents at the observational level by Understanding Society, with no weight at the person level. The analysis was repeated using three different methods for partitioning this observation level weight into a person level and observation level weight. Alternative method A corresponds to method A in Carle, 2009 [1] and method 2 in Rabe-Hesketh & Skrondal, 2006 [2]. Alternative method B corresponds to method B in Carle, 2009 and method 1 in Rabe-Hesketh & Skrondal, 2006. Weighting method C used a survey design enumeration weight at the person level, combined with the observation level weight from the main analyses.

Table S4 Results of a mixed effects generalised linear model comparing the total SDQ scores between different groups of children aged 5 and 8 in the UK Longitudinal Household Study main survey or 5-11 in the COVID-19 surveys, before and during the pandemic, weighted using four alternative weight scaling mechanism.

	**Weights used in main analysis**		**Alternative weighting method A**		**Alternative weighting method B**		**Alternative weighting method C**	
**Characteristic**	**Difference in SDQ before the pandemic**	**Difference in SDQ during the pandemic**	**Difference in SDQ before the pandemic**	**Difference in SDQ during the pandemic**	**Difference in SDQ before the pandemic**	**Difference in SDQ during the pandemic**	**Difference in SDQ before the pandemic**	**Difference in SDQ during the pandemic**
Female (ref: Male)	-1.29 (-1.64 to -0.93)	-1.02 (-1.66 to -0.37)	-1.20 (-1.62 to -0.77)	-0.74 (-1.50 to -0.03)	-1.22 (-1.65 to -0.80)	-0.86 (-1.60 to -0.11)	-1.32 (-1.73 to -0.92)	-0.87 (-1.91 to +0.17)
Non-white ethnicity (ref: White)	-0.76 (-1.13 to -0.39)	-1.28 (-1.99 to -0.58)	-1.26 (-1.71 to -0.81)	-1.94 (-2.83 to -1.05)	-1.23 (-1.66 to -0.80)	-1.83 (-2.68 to -0.98)	-1.00 (-1.42 to -0.57)	-1.90 (-3.08 to -0.72)
Single parenting (ref: Couple parenting)	+1.58 (+1.09 to +2.08)	+0.86 (-0.17 to +1.90)	+1.58 (+1.08 to +2.09)	+0.82 (-0.34 to +1.98)	+1.56 (+1.06 to +2.07)	+0.81 (-0.34 to +1.96)	+1.58 (+1.02 to +2.14)	+1.36 (+0.08 to +2.64)
Parent highest education below degree (ref: Degree)	+1.95 (+1.60 to +2.31)	+1.21 (+0.54 to +1.88)	+2.14 (+1.70 to +2.58)	+1.41 (+0.52 to +2.31)	+2.10 (+1.69 to +2.51)	+1.35 (+0.57 to +2.13)	+2.00 (+1.60 to +2.39)	+1.12 (+0.01 to +2.23)
Parents unemployed (ref: At least one employed parent)	+2.35 (+1.72 to +2.98)	+0.02 (-1.10 to +1.13)	+2.39 (+1.75 to +3.04)	+0.10 (-1.10 to +1.29)	+2.30 (+1.65 to +2.94)	+0.00 (-1.16 to +1.17)	+2.51 (+1.80 to +3.23)	+0.21 (-1.11 to +1.53)
Low household income (ref: High household income)	+0.88 (+0.40 to +1.36)	+0.22 (-0.62 to +1.05)	+0.81 (+0.33 to +1.30)	+0.09 (-0.79 to +0.98)	+0.80 (+0.31 to +1.29)	+0.09 (-0.79 to +0.97)	+1.04 (+0.48 to +1.61)	+0.12 (-0.80 to +1.05)
High deprivation area (ref: Low deprivation area)	+1.56 (+1.12 to +2.00)	+1.83 (+1.00 to +2.67)	+1.51 (+1.01 to +2.02)	+1.83 (+0.84 to +2.82)	+1.47 (+0.97 to +1.96)	+1.76 (+0.82 to +2.70)	+1.39 (+0.89 to +1.89)	+2.02 (+0.63 to +3.40)

Supplementary file 1-5. Description of the sample characteristics at child level and person-year level

Table S5: Description of the sample at a child level and person-year level before and after weighting, overall and stratified into those measured before and those measured during the COVID-19 pandemic, alongside the 2011-2012 weighted sample (where attrition is lowest).

Characteristic	2011-2012 weighted sample %	Before the COVID-19 pandemic[a]			During the COVID-19 pandemic[b]			Total		
		Unweighted Number of children (%)	Unweighted Person-years (%)	Weighted person-years %	Unweighted Number of children (%)	Unweighted Person-years (%)	Weighted person-years %	Unweighted Number of children (%)	Unweighted Person-years (%)	Weighted person-years %
**Sex**										
Male	52.9	4,430 (51.5)	6,101 (51.4)	50.9	1,038 (51.6)	2,331 (52.5)	49.6	4,755 (51.7)	8,432 (51.7)	50.5
Female	47.1	4,168 (48.5)	5,766 (48.6)	49.1	973 (48.4)	2,113 (47.6)	50.4	4,446 (48.3)	7,879 (48.3)	49.5
**Ethnicity**										
White	83.5	6,027 (71.4)	8,336 (71.4)	82.5	1,480 (72.8)	3,306 (74.0)	82.6	6,416 (70.7)	11,642 (72.1)	82.6
Asian	6.2	1,137 (13.5)	1,567 (13.4)	6.3	212 (10.4)	418 (9.4)	4.5	1,249 (13.8)	1,985(12.3)	5.7
Black	3.5	410 (4.9)	553 (4.7)	2.9	52 (2.6)	98 (2.2)	1.2	442 (4.9)	651 (4.0)	2.3
Mixed	6.0	813 (9.6)	1,137 (9.7)	7.7	275 (13.5)	620 (13.9)	11.3	903 (10.0)	1,757 (10.9)	8.8
Other	0.8	59 (0.7)	84 (0.7)	0.6	15 (0.7)	28 (0.6)	0.5	69 (0.8)	112 (0.7)	0.6
**Lone parent**	23.8	1,818 (21.4)	2,306 (19.6)	22.1	292 (14.3)	527 (11.7)	17.8	1,974 (21.6)	2,833 (17.4)	20.7
**Highest parent education**										
Degree	48.0	4,135 (51.6)	5,773 (52.1)	51.7	1,167 (60.7)	2601 (61.1)	53.5	4,499 (52.3)	8,374 (54.6)	52.3
Upper secondary (A-level)	9.0	758 (9.5)	1,067 (9.6)	9.3	197 (10.3)	443 (10.4)	9.6	821 (9.6)	1,510 (9.9)	9.4
Lower secondary (GCSE)	30.5	2,021 (25.2)	2,743 (24.8)	27.7	338 (17.6)	730 (17.2)	27.6	2,111 (24.6)	3,473 (22.7)	27.7
None	12.5	1,098 (13.7)	1,493 (13.5)	11.3	220 (11.5)	481 (11.3)	9.4	1,167(13.6)	1,974 (12.9)	10.7
**No parent employed[e]**	19.2	1,536 (18.7)	1,819 (16.2)	15.9	304 (14.9)	491 (10.9)	14.9	1,757 (19.8)	2,310 (14.7)	15.6
**Low-income household**	15.3	1,505 (20.3)	1,695 (16.7)	15.9	608 (29.7)	850 (18.9)	21.7	2,013 (24.5)	2,545 (17.4)	17.8
**Resident in high deprivation area**	22.1	2,190 (25.5)	2,884 (24.4)	22.0	362 (16.0)	669 (15.0)	16.5	2,324 (25.2)	3,553 (21.8)	20.2
**Mean age (SD)**	6.4 (1.4)	6.5 (1.5)		6.5 (1.4)	8.2 (1.9)		8.2 (1.7)	7.0 (1.8)		7.0 (1.7)
**Mean SDQ score (SD)**	8.6 (5.7)	8.5 (5.9)		8.7 (5.9)	8.8 (6.2)		9.6 (6.8)	8.6 (6.0)		9.0 (6.2)
**Total N**	**1,751**	**8,598**	**11,868**	**10,680[c]**	**2,046**	**4,493**	**4,073[c]**	**9,272**	**16,361**	**15,114[c]**

[a] Understanding Society surveys between 2011-2019
[b] Understanding Society COVID surveys in July 2020, September 2020, March 2021.
[c] Person -years contributing to the weighted proportions.

Supplementary file 1-6. Trends in cross-sectional average SDQ score among 8-year-olds

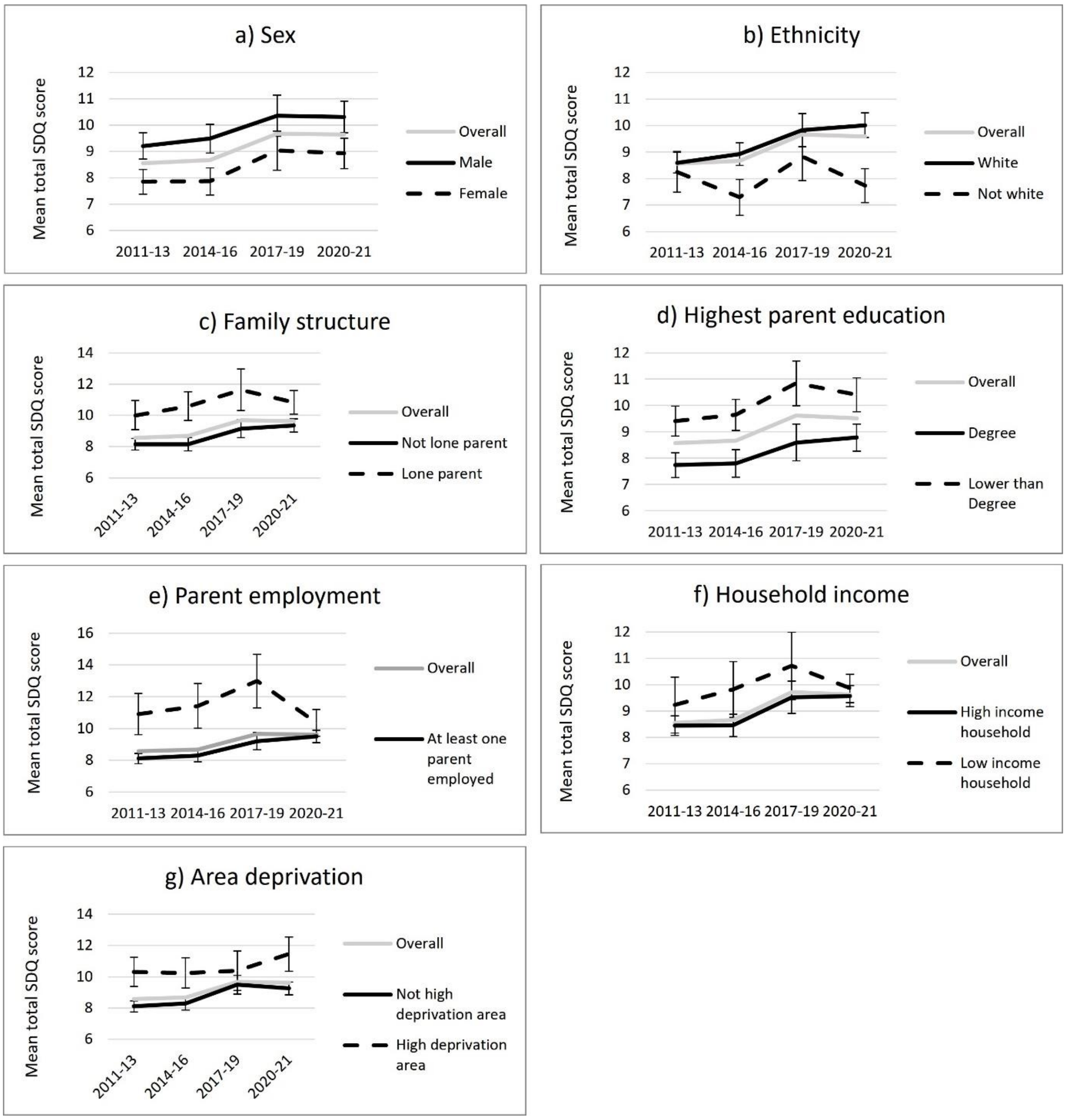

Figure S3 Trends in the total SDQ score, representing severity of mental health symptoms, amongst 8 year olds in the UK between 2011 and 2021. SDQ scores are stratified by seven measures of inequality: a) sex (male or female) b) ethnicity (white or not white) c) family structure (single or coupled parenting) d) highest parent education (degree or lower) e) parent employment (at least one parent employed or no parent employed) f) household income (less than 60% of the median that year or higher) g) area deprivation (resident in the 20% most deprived areas in that country or not). The vertical bars show the 95% confidence intervals. Each graph includes all participants with a SDQ score and complete data on the relevant inequality, weighted for survey design and non-response.

Supplementary file 1-7. Exploring sensitivity to categorisations of area deprivation and higher education

Supplementary file 1-7.1. Alternative categorisations of area deprivation

Table S6 Results of a mixed effects generalised linear model comparing SDQ scores between children aged 5 and 8 in the UK Longitudinal Household Study main survey or 5-11 in the COVID-19 surveys, who live in more or less deprived areas, under different categorisations of more deprived areas, both before and during the pandemic.

	**Difference in SDQ before the pandemic**	**Difference in SDQ during the pandemic**
Resident in 20% most deprived areas in each UK country (ref: 80% least deprived areas)	+1.56 (+1.12 to +2.00)	+1.83 (+1.00 to +2.67)
Resident in 80% most deprived areas in each UK country (ref: 20% least deprived areas)	+1.82 (+1.42 to +2.22)	+1.14 (+0.42 to +1.86)

Supplementary file 1-7.2. Alternative categorisations of higher education

Table S7 Results of a mixed effects generalised linear model comparing SDQ scores between aged 5 and 8 in the UK Longitudinal Household Study main survey or 5-11 in the COVID-19 surveys, whose parents have higher or lower educational attainment, under different categorisations of higher education, both before and during the pandemic.

	**Difference in SDQ before the pandemic**	**Difference in SDQ during the pandemic**
(Both) Parent highest education below degree (ref: Degree)	+1.95 (+1.60 to +2.31)	+1.21 (+0.54 to +1.88)
(Both) Parent highest education lower than secondary (GCSE) (ref: Lower secondary (GCSE) or higher)	+0.90 (+0.30 to +1.50)	+0.23 (-0.92 to +1.37)

Supplementary file 1-8. Analysis of externalised and internalised child mental health symptoms

Table S8 Results of a mixed effects generalised linear model comparing the total, externalising and internalising SDQ scores between different groups of children aged 5 and 8 in the UK Longitudinal Household Study main survey or 5-11 in the COVID-19 surveys, before and during the pandemic.

	**Total SDQ score**		**Externalising SDQ score**		**Internalising SDQ score**	
**Characteristic**	**Difference in SDQ before the pandemic**	**Difference in SDQ during the pandemic**	**Difference in SDQ before the pandemic**	**Difference in SDQ during the pandemic**	**Difference in SDQ before the pandemic**	**Difference in SDQ during the pandemic**
Female (ref: Male)	-1.29 (-1.64 to -0.93)	-1.02 (-1.66 to -0.37)	-1.20 (-1.41 to -0.98)	-1.13 (-1.52 to -0.75)	-0.12 (-0.32 to +0.08)	+0.10 (-0.28 to +0.48)
Non-white ethnicity (ref: White)	-0.76 (-1.13 to -0.39)	-1.28 (-1.99 to -0.58)	-0.71 (-0.94 to -0.48)	-0.90 (-1.32 to -0.48)	-0.05 (-0.26 to +0.16)	-0.36 (-0.78 to +0.07)
Single parenting (ref: Couple parenting)	+1.58 (+1.09 to +2.08)	+0.86 (-0.17 to +1.90)	+0.88 (+0.58 to +1.17)	+0.56 (-0.05 to +1.17)	+0.76 (+0.46 to +1.06)	+0.40 (-0.22 to +1.03)
Parent highest education below degree (ref: Degree)	+1.95 (+1.60 to +2.31)	+1.21 (+0.54 to +1.88)	+1.18 (+0.96 to +1.40)	+0.86 (+0.46 to +1.27)	+0.79 (+0.59 to +0.99)	+0.34 (-0.05 to +0.74)
Parents unemployed (ref: At least one employed parent)	+2.35 (+1.72 to +2.98)	+0.02 (-1.10 to +1.13)	+1.06 (+0.69 to +1.43)	+0.23 (-0.42 to +0.88)	+1.37 (+0.98 to +1.76)	-0.10 (-0.75 to +0.56)
Low household income (ref: High household income)	+0.88 (+0.40 to +1.36)	+0.22 (-0.62 to +1.05)	+0.42 (+0.13 to +0.70)	+0.07 (-0.41 to +0.55)	+0.45 (+0.17 to +0.74)	+0.19 (-0.32 to +0.69)
High deprivation area (ref: Low deprivation area)	+1.56 (+1.12 to +2.00)	+1.83 (+1.00 to +2.67)	+0.82 (+0.55 to +1.09)	+1.08 (+0.59 to +1.58)	+0.75 (+0.49 to +1.02)	+0.79 (+0.22 to +1.37)

Supplementary file 1-9. Analysis of proportion of children with abnormal SDQ score

Table S9 Results of a mixed effects Poisson regression model comparing risk of experiencing poor mental health between different groups of children aged 5 and 8 in the UK Longitudinal Household Study main survey or 5-11 in the COVID-19 surveys, before and during the pandemic. The analysis was repeated where the binary outcome (poor mental health) was first defined as a borderline or abnormal SDQ score (>13) and then as an abnormal SDQ score (>16).

	**Binary risk of experiencing borderline or abnormal mental health scores (SDQ>13)**		**Binary risk of experiencing abnormal mental health scores (SDQ>16)**	
**Characteristic**	**Relative risk before the pandemic**	**Relative risk during the pandemic**	**Relative risk before the pandemic**	**Relative risk during the pandemic**
Female (ref: Male)	0.77 (0.39-1.50)	1.05 (0.76-1.45)	0.89 (0.35-2.24)	0.97 (0.63-1.49)
Non-white ethnicity (ref: White)	0.78 (0.66-0.92)	0.61 (0.42-0.88)	0.64 (0.50-0.83)	0.55 (0.32-0.95)
Single parenting (ref: Couple parenting)	1.75 (1.52-2.02)	1.39 (1.02-1.92)	1.73 (1.42-2.10)	1.75 (1.13-2.70)
Parent highest education below degree (ref: Degree)	1.77 (1.54-2.03)	1.40 (1.07-1.83)	2.08 (1.73-2.51)	1.42 (0.99-2.04)
Parents unemployed (ref: At least one employed parent)	2.18 (1.88-2.52)	1.38 (1.00-1.90)	2.65 (2.18-3.22)	1.68 (1.12-2.53)
Low household income (ref: High household income)	1.42 (1.23-1.64)	1.13 (0.85-1.51)	1.38 (1.14-1.67)	1.25 (0.86-1.81)
High deprivation area (ref: Low deprivation area)	1.37 (1.17-1.60)	1.79 (1.30-2.45)	1.68 (1.38-2.04)	1.96 (1.33-2.90)

Bibliography

1. Carle, A., (2009.) Fitting multilevel models in complex survey data with design weights: Recommendations. *BMC Medical Research Methodology,* **9**.
2. Rabe-Hesketh, S. and Skrondal, A., (2006.) Multilevel modelling of complex survey data. *Journal of the Royal Statistical Society: Series A (Statistics in Society),* **169**(4): p. 805-827.
